# Piecing Arterial Branching Pattern Together from Non-Contrast and Angiographic Brain Computed Tomography before Endovascular Thrombectomy for Acute Ischemic Stroke

**DOI:** 10.3390/jcm12124051

**Published:** 2023-06-14

**Authors:** Horyul Lee, Woojin Shim, Dongjun Jeong, Younghoon Kwon, Sung Won Youn

**Affiliations:** 1 Department of Radiology, Daegu Catholic University Medical Center, Daegu 42472, Republic of Korea; dlghfuf1234@naver.com (H.L.);; 2 Department of Cardiology, University of Washington School of Medicine, Seattle, WA 98195, USA; 3 Department of Radiology, Daegu Catholic University Medical School, Daegu 42472, Republic of Korea

**Keywords:** Cerebral Arteries, Cerebrovascular Occlusion, Aspiration Thrombectomy, Acute Ischemic Stroke, Computed Tomography, CT Angiography

## Abstract

Predicting the unseen arterial course and branching pattern distal to vessel occlusion is crucial for endovascular thrombectomy in acute stroke patients. We investigated whether a comprehensive interpretation of NCT and CTA would enhance arterial course prediction more than either NCT or CTA interpretation alone. Among 150 patients who achieved post-thrombectomy TICI grades ≥ IIb for anterior circulation occlusions, we assessed visualization grade on both NCT and CTA by five scales at the thrombosed and the distal-to-thrombus segment, using DSA as the reference standard. The visualization grades were compared and related to various subgroups. The mean visualization grade of the distal-to-thrombus segment on NCT was significantly larger than that of CTA (mean ± SD, 3.62 ± 0.87 versus 3.31 ± 1.20; *p* < 0.05). On CTA, visualization grade of distal-to-thrombus segment in the good collateral flow subgroup was higher than that in the poor collateral flow subgroup (mean ± SD, 4.01 ± 0.93 versus 2.56 ± 0.99; *p* < 0.001). After the comprehensive interpretation of NCT and CTA, seventeen cases (11%) showed visualization grade of distal-to-thrombus segment upgrading. Tracing arterial course and piecing branching patterns together in distal-to-occlusion of stroke patients was feasible on the routine pre-interventional NCT and CTA, which may provide timely guidance during thrombectomy.

## 1. Introduction

Endovascular thrombectomy has gained the status of “gold standard of treatment” for acute ischemic stroke with large vascular occlusion. Since landmark clinical trials have proved its efficacy, endovascular thrombectomy has been increasingly performed to treat acute intracranial artery occlusion around the world [1,2,3]. The presence of a thrombus, the target of retrieval, can be detected by the hyperattenuating arterial sign on a non-contrast computed tomography (CT) before thrombectomy [4,5]. Besides detecting the thrombus segment, it is critical to predict the course of the distal-to-thrombus segment before thrombectomy. Predicting the unseen arterial course and branching pattern distal to the occlusion site may help the operator select appropriate devices and guide them during the thrombectomy. It is also necessary to delineate the overall course of an occluded vessel, including the distal-to-thrombus segment, because the catheter and wire advancing into the wrong pathway might result in complications such as subarachnoid hemorrhage, which can lead to devastating results [6]. However, it cannot be visualized on fluoroscopy during thrombectomy, forcing the operator to perform the procedure blindly beyond the thrombus segment. 

Although several approaches have been taken to visualize the distal-to-thrombus segment before thrombectomy using various sequences of magnetic resonance imaging (MRI) [7,8,9,10,11,12] and intra-arterial cone-beam CT [13,14,15], no effort has been made to visualize the distal-to-thrombus segment using non-contrast (NCT) and angiographic brain CT (CTA), which is a routine imaging protocol to triage acute stroke patients. Although NCT can detect thrombus by hyperdense arterial signs, it remains unknown whether NCT can track the arterial trajectory distal to the occlusion site in a patient with acute ischemic stroke. In this retrospective single-center study in patients who underwent endovascular thrombectomy due to acute ischemic stroke with large vessel occlusion, we investigated whether (1) a distal-to-thrombus segment of an occluded artery can be traced either on NCT or CTA before thrombectomy, and (2) whether a comprehensive interpretation of NCT and CTA would enhance arterial course prediction more than either NCT or CTA interpretation alone. This feasibility study assessed and compared arterial visualization grade of a thrombosed segment and distal-to-thrombus segment of an occluded artery on NCT and CTA.

## 2. Materials and Methods

The study was conducted according to the guidelines of the Declaration of Helsinki, and approved by the Institutional Review Board of Daegu Catholic University Hospital (CR-22-156-L, 17 November 2022). The patient consents were waived due to the retrospective study design. 

### 2.1. Patients

**Figure 1:**
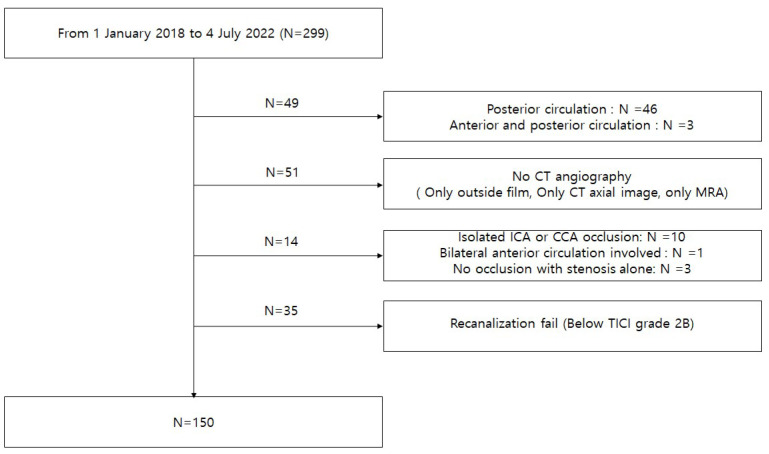
Flow chart for patient selection. A total of 150 patients were enrolled who underwent endovascular thrombectomy for anterior circulation large-vessel occlusions and achieved post-thrombectomy TICI grades ≥ IIb. CT, computed tomography; MRA, magnetic resonance angiography; ICA, internal carotid artery; CCA, common carotid artery; TICI, thrombolysis in cerebral infarction.

From the hospital information system, the patients who underwent endovascular thrombectomy for acute ischemic stroke with large vessel occlusion of the anterior circulation were included in this study. Between January 2018 and July 2022, we treated 299 patients in our center due to acute ischemic stroke (Figure 1 and Table 1). We excluded cases with posterior circulation (*n* = 49), cases without CTA (*n* = 51), cases with isolated internal carotid artery (ICA) or common carotid artery (CCA) occlusion (*n* = 10), cases with bilateral anterior circulation involvement (*n* = 1), cases without occlusion but with stenosis alone (*n* = 3), and cases that did not recanalize (*n* = 35). A total of 150 patients met the eligibility criteria. The median age of the patients was 73 (ranging from 20 to 95). The number of male patients was 84 (56%). The occlusion sites were classified as (1) ICA terminus occlusion (*n* = 15; 10%), (2) ICA and MCA tandem occlusion (*n* = 48; 32%), (3) M1 occlusion (*n* = 75; 50%), and (4) M2 occlusion (*n* = 12; 8%). The initial National Institutes of Health Stroke Scale (NIHSS) and modified Rankin Scale (mRS) were 13 ± 5 and 0.2 ± 0.8, respectively. The medical conditions present included hypertension (*n* = 84; 56%), diabetes mellitus (*n* = 36; 24%), hyperlipidemia (*n* = 52; 34.7%), and cardiac-originated emboli (*n* = 63; 42%). Discharge NIH and mRS were 10.5 ± 9.3 and 4 ±1.4, respectively. The obtained CT imaging protocols included cerebral artery CTA (*n* = 82; 54.7%), perfusion CT and CTA (*n* = 66; 44%), and carotid artery CTA (*n* = 2; 1.3%).

### 2.2. CT Scanning Protocols

Parameters of the non-enhanced brain CT were as follows: tube voltage: 120 kVp, tube current: 330 mAs, detector coverage: 40 mm, beam pitch: 0.516:1, coverage speed: 34.38 mm/s, rotation time: 0.6 s. Parameters of the cerebral artery CTA were as follows: tube voltage: 100 kVp, tube current: 380 mAs, detector coverage: 40 mm, beam pitch: 0.516:1, coverage speed: 41.25 mm/s, rotation time: 0.5 s, IV contrast 4.5 ml/s with 90 mL and IV saline 4.5 ml/s with 30 mL. Parameters of brain perfusion CT were as follows: tube voltage: 80 kVp, tube current: 200 mAs, scan type: axial, detector coverage: 160 mm, rotation time: 0.5 s, number of passes: 20, IV contrast 5 ml/s with 60 mL and IV saline 5 ml/s with 40 mL. Parameters of the carotid artery CTA were as follows: tube voltage: 100 kVp, tube current: 150–600 mAs, detector coverage: 40 mm, beam pitch: 0.984:1, coverage speed: 65.63 mm/s, rotation time: 0.6 s, IV contrast 4.5 ml/s with 70 mL and IV saline 5 ml/s with 40 mL. The maximal intensity projection (MIP) images of CTA were reconstructed as three planes of axial, coronal, and sagittal in a 5 mm thickness. 

### 2.3. Image Analysis

**Figure 2:**
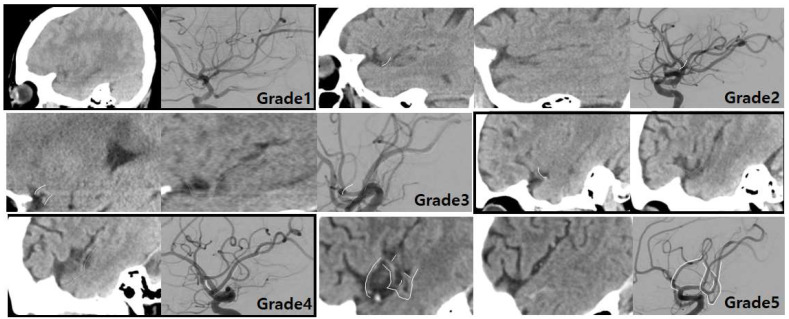
NCT grading of distal-to-thrombus segment in correspondence to the reference standard of final DSA findings. NCT showed no MCA course at all (grade 1), only a small portion of the inferior division of MCA (grade 2), almost all portions of the inferior division of MCA (grade 3), a small portion of the inferior division and almost all of the superior division of MCA (grade 4), and all of the inferior division and superior division of MCA (grade 5), respectively. The arterial courses on NCT were matched with those on DSA as indicated by solid and dotted lines. NCT, non-contrast computed tomography; DSA, digital subtraction angiography; MCA, middle cerebral artery.

**Figure 3:**
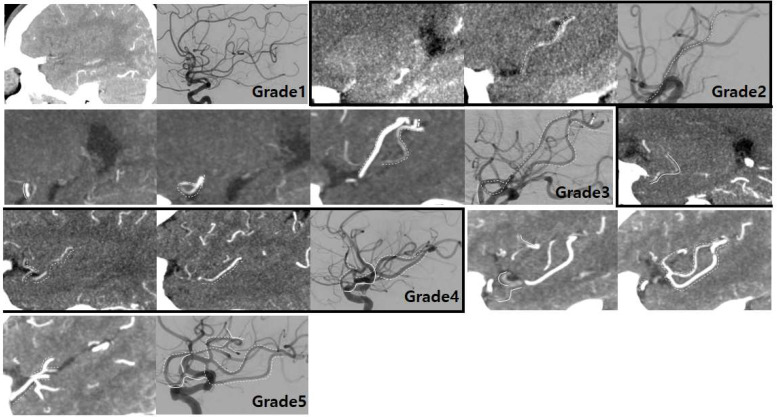
CTA grading of distal-to-thrombus segment in correspondence to the reference standard of final DSA findings. CTA showed no MCA course at all (grade 1), only a small portion of the inferior division of MCA (grade 2), only a small portion of the superior and inferior division of MCA (grade 3), almost all of the inferior division and small portion of the superior division of MCA (grade 4), and all of the inferior division and superior division of MCA (grade 5), respectively. The arterial courses on CTA were matched with those on DSA as indicated by solid (MCA proximal portion) and dotted lines (MCA distal portions). CTA, computed tomography angiography; DSA, digital subtraction angiography; MCA, middle cerebral artery.

All images were evaluated by one interventional neuroradiologist with 20 years’ experience and a trainee with 3 years’ experience. We reviewed the CT images on a picture archiving and communication system and scored arterial visualization grade on the sagittal plane in consensus. By using a 5-point scale, we assessed the visualization grade of the arteries in the thrombosed segment and the overall course of occluded intracranial artery, including vessels of the distal-to-thrombus segment in NCT and CTA (Supplementary Table S1; Figure 2 and Figure 3).

The collateral score was assessed as from 0 to 4, depending on the modified system of Yuan et al. [16]. Collateral scores were based on digital subtraction angiography (DSA). We defined a collateral score of 0 to 4 as follows in Supplementary Table S2. We defined a collateral score of 0 to 2 as poor collateral and 3 to 4 as good collateral (Supplementary Figure S1). 

However, when collateral scoring was impossible, for example, in the case of proximal ICA blockage, spontaneous recanalization, or distal migration of initial thrombus on DSA, CTA collateral scoring was used.

### 2.4. Statistical Analysis 

Using a paired *t*-test, we compared the NCT grade and the CTA grade for the thrombosed segment and the distal-to-thrombus segment, respectively. Using an independent two-sample *t*-test, we compared (1) the CTA grade of a distal-to-thrombus segment of a good collateral subgroup and that of a poor collateral subgroup, (2) the CTA grade of a distal-to-thrombus segment of a contrast medium permeation subgroup and that of a non-permeation subgroup. 

We compared the diagnostic performance of NCT and CTA to determine the occlusion side hemisphere using the receiver operating characteristic curve analysis. We counted cases where the grade of a distal-to-thrombus segment improved on comprehensive interpretation. The statistical analysis was performed with the software SPSS (version 28.0; IBM Corp., Armonk, NY, USA). The variables showing *p* values < 0.05 were considered statistically significant.

## 3. Results

### 3.1. Arterial Visualization Grade of Thrombosed Segment 

**Figure 4:**
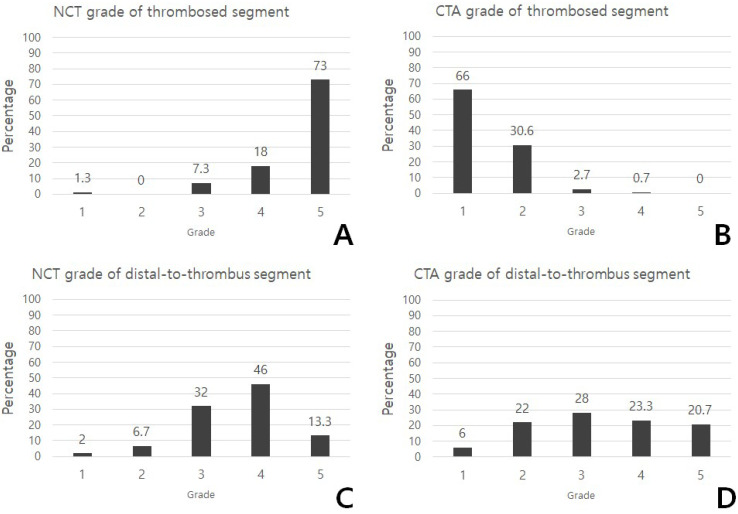
Distribution of grade of thrombosed segments and distal-to-thrombus segments in NCT and CTA. Incidence of NCT grade of thrombosed segments 1–5 (**A**), CTA grade of thrombosed segments 1–5 (**B**), NCT grade of distal-to-thrombus segments 1–5 (**C**), and CTA grade of distal-to-thrombus segments 1–5 (**D**) among a total of 150 patients. NCT, non-contrast computed tomography; CTA, computed tomographic angiography.

Among the total number of 150 patients, the grade of thrombosed segments was 4.62 ± 0.74 (mean ± SD) on NCT and 1.38 ± 0.58 on CTA, respectively (Figure 4). The NCT grade of thrombosed segments showed 2 cases of grade 1 (1.3%), 0 of grade 2 (0%), 11 of grade 3 (17.3%), 27 of grade 4 (18.0%), and 110 of grade 5 (73.3%). The CTA grade of thrombosed segments showed 99 cases of grade 1 (66%), 46 of grade 2 (30.7%), 4 of grade 3 (2.7%), 1 of grade 4 (0.7%), and 0 of grade 5. 

### 3.2. Arterial Visualization Grade of Distal-to-Thrombus Segment 

The grade of distal-to-thrombus segments was 3.62 ± 0.87 (mean ± SD) on NCT and 3.31 ± 1.20 on CTA, respectively. Among the total number of 150 patients, the NCT grade of distal-to-thrombus segments showed 3 cases of grade 1 (2%), 10 cases of grade 2 (6.7%), 48 cases of grade 3 (32%), 69 cases of grade 4 (46.0%), and 20 cases of grade 5 (13.3%). The CTA grade of distal-to-thrombus segments showed 9 cases of grade 1 (6%), 33 cases of grade 2 (22%), 42 cases of grade 3 (28%), 35 cases of grade 4 (23.3%), and 31 cases of grade 5 (20.7%).

### 3.3. Comparison of Arterial Visualization Grades on NCT with Those on CTA 

The mean grade of the thrombosed segments on NCT was significantly larger than the mean grade of the thrombosed segments on CTA (mean ± SD, 4.62 ± 0.74 vs. 1.38 ± 0.58; *p* < 0.001). The mean grade of the distal-to-thrombus segments on NCT was significantly larger than the mean grade of distal-to-thrombus segments on CTA (mean ± SD, 3.62 ± 0.87 vs. 3.31 ± 1.20; *p* < 0.05). Among the total of 150 patients, 110 patients (73%) showed an NCT grade of the distal-to-thrombus segment that was equal to or higher than the CTA grade of the distal-to-thrombus segment, while 40 patients (27%) showed a CTA grade of the distal-to-thrombus segment that was higher than the NCT grade of the distal-to-thrombus segment.

### 3.4. Collateral Flow Score and Arterial Visualization Grade

**Figure 5:**
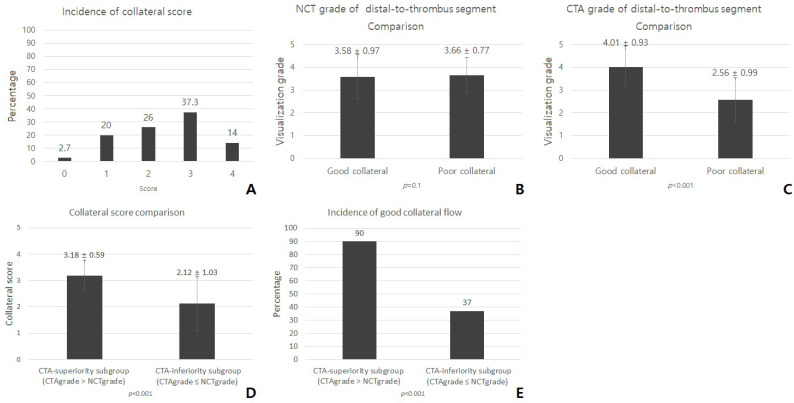
Collateral grade and visualization grade of distal-to-thrombus segments on NCT and CTA. (**A**) Incidence of collateral grade. (**B**) The NCT grade of distal-to-thrombus segment in the good collateral subgroup was not significantly different from that in the poor collateral flow subgroup. (**C**) The CTA grade of the distal-to-thrombus segment in the good collateral subgroup was higher than that in the poor collateral flow subgroup. (**D**) The mean collateral grade of the CTA-superiority subgroup (CTA_grade_ > NCT_grade_) was higher than that of the CTA-inferiority subgroup (CTA_grade_ ≤ NCT_grade_). (**E**) The incidence of good collateral flow in the CTA-superiority subgroup (CTA_grade_ > NCT_grade_) was higher than that in the CTA-inferiority subgroup (CTA_grade_ ≤ NCT_grade_). NCT, non-contrast computed tomography; CTA, computed tomographic angiography.

The collateral score results showed 4 cases of score 0 (2.7%), 30 of score 1 (20%), 39 of score 2 (26%), 56 of score 3 (37.3%), and 21 of score 4 (14%) (Figure 5). 

The NCT grade of the thrombosed segment in the good collateral flow subgroup was 4.46 ± 0.92 (mean ± SD), while that in the poor collateral flow subgroup was 4.78 ± 0.45 (mean ± SD; *p* < 0.001). 

The CTA grade of the thrombosed segment in the good collateral flow subgroup was 1.39 ± 0.54 (mean ± SD), while that in the poor collateral flow subgroup was 1.36 ± 0.61 (mean ± SD; *p* = 0.925). 

The NCT grade of the distal-to-thrombus segment in the good collateral flow subgroup was 3.58 ± 0.97 (mean ± SD), while that in the poor collateral flow subgroup was 3.66 ± 0.77 (mean ± SD; *p* = 0.1).

The CTA grade of the distal-to-thrombus segment in the good collateral flow subgroup was higher than that in the poor collateral flow subgroup (mean ± SD, 4.01 ± 0.93 vs. 2.56 ± 0.99; *p* < 0.001). 

In terms of the distal-to-thrombus segment, the mean collateral score of the CTA-superiority subgroup (CTA_grade_ > NCT_grade_) was higher than that of the CTA-inferiority subgroup (CTA_grade_ ≤ NCT_grade_) (mean ± SD, 3.18 ± 0.59 vs. 2.12 ± 1.03; *p* < 0.001). The incidence of good collateral flow in the CTA-superiority subgroup (CTA_grade_ > NCT_grade_) was higher than that in the CTA-inferiority subgroup (CTA_grade_ ≤ NCT_grade_) (mean ± SD, 90% (36/40) vs. 37% (41/110); *p* < 0.001).

### 3.5. CTA Grade of Thrombosed Segment and CTA Grade of Distal-to-Thrombus Segment

The contrast medium permeation subgroup was defined as the CTA grade of the thrombosed segments from 2 to 5. The non-permeation subgroup was defined as the CTA grade of thrombosed segment 1. The CTA grade in the distal-to-thrombus segment of the permeation subgroup was higher than that of the non-permeation subgroup (mean ± SD, 3.73 ± 1.22 vs. 3.09 ± 1.14; *p* < 0.01).

### 3.6. Comparison between the Cardiac Embolic Subgroup and Non-Cardiac Embolic Subgroup

Between the cardiac embolic and non-cardiac embolic subgroups, no visualization difference was found in the NCT grade of thrombosed segment (4.63 ± 0.63 vs. 4.61 ± 0.81 (*p* = 0.436)), the CTA grade of thrombosed segment (1.38 ± 0.52 vs. 1.38 ± 0.62 (*p* = 0.51)), the NCT grade of distal-to-thrombus segment (3.63 ± 0.89 vs. 3.61 ± 0.87 (*p* = 0.99)), and the CTA grade of distal-to-thrombus segment (3.35 ± 1.1 vs. 3.28 ± 1.27 (*p* = 0.07)), respectively.

### 3.7. NCT and CTA Performance in Detecting the Occlusion Side

Either on NCT or CTA, diagnostic performance to identify the occluded side was evaluated using the arterial visualization grade of the thrombosed segment and distal-to-thrombus segment, respectively (Supplementary Figure S2). The CTA grade of the distal-to-thrombus segment and the CTA grade of the thrombosed segment had areas under the curves of 1.00 and 0.897, respectively. The NCT grade of the distal-to-thrombus segment and the NCT grade of the thrombosed segment had respective areas under the curves of 0.498 and 0.535, respectively.

### 3.8. A Comprehensive Reading of NCT and CTA

**Figure 6:**
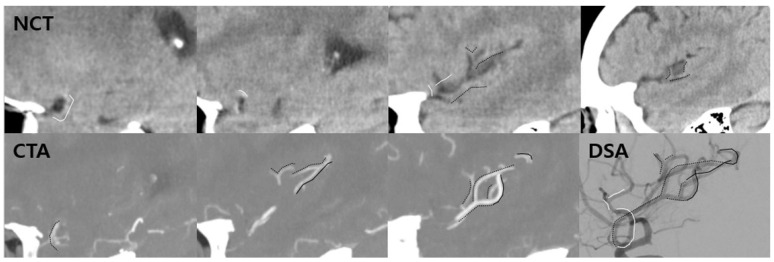
Upscaling of arterial visualization grade after comprehensive reading. A patient with MCA occlusion shows NCT grade 3 of distal-to-thrombus segment and CTA grade 3 of distal-to-thrombus segment. After comprehensive reading, the arterial visualization grade of the distal-to-thrombus segment was 5. (NCT) A small portion of the inferior division and superior division of MCA was visible on NCT. (CTA) Almost all the portion of the inferior division and no superior division was visible on CTA. (DSA) Piecing arterial course together from NCT and CTA constitutes the entire MCA branching pattern. White line = arterial course visualized on NCT only; black dotted line = visualized both on NCT and CTA; black solid line = visualized on CTA only. MCA, middle cerebral artery; NCT, non-contrast computed tomography; CTA, computed tomography angiography; DSA, digital subtraction angiography.

The comprehensive interpretation of NCT and CTA showed 17 cases (11%) among 150 patients had an upgrade of the arterial visualization grade of distal-to-thrombus segment (4.29 ± 0.47, mean ± SD) (Figure 6 and Supplementary Table S3).

## 4. Discussion

The dual task of detecting the site of large vessel occlusions and further designing endovascular thrombectomy in acute ischemic stroke patients requires precise pre-procedural imaging interpretation and guidance. An unknown arterial course distal to the occlusion site poses a challenge in the thrombectomy of acute stroke cases with large vessel occlusion. Blind advancing of endovascular devices can result in arterial perforation or subarachnoid hemorrhage, leading to devastating outcomes [6]. Perforations most commonly occur at the distal-to-thrombus segment while traversing the occlusion site with a microcatheter or microwire, or while withdrawing a stent retriever. The trajectory of the distal-to-thrombus segment can be assumed only by an experienced operator, who relies on fluoroscopic-guided curvature of the radiopaque devices passing through the occluded segment. There may be hidden traps behind the thrombotic occlusion such as unfavorable arterial anatomy including curved MCA, [17,18] variations in MCA branching pattern, [19,20,21] and the presence of cerebral aneurysms [22,23]. This uncertainty of the vascular trajectory makes thrombectomy itself technically challenging and prolongs procedure time. This increases the risk of revascularization failure, particularly in the setting of markedly curved branched arteries of MCA [17,18]. Endovascular thrombectomy fails in approximately 20% of all cases, and about 20% of such thrombectomy failures are due to the inability of devices to access or pass through a thrombus [24]. Therefore, understanding the underlying vascular anatomy is crucial to successful thrombectomy. On the other hand, it is known that patients with acute ischemic stroke who are eligible for thrombectomy have a higher prevalence of cerebral aneurysms (3.7–5.6%) than a healthy reference population [22,23]. Therefore, in case of a cerebral aneurysm being present in the distal-to-thrombus segment near bifurcations, blind device advancement may be complicated by intra-procedural perforations.

To overcome this challenge, there have been efforts to visualize the distal-to-thrombus segment before thrombectomy using various sequences of MRI, [7,8,9,10,11,12] or intra-arterial cone-beam CT angiography [13,14,15]. Arterial structural anatomy can be shown by the time of flight-magnetic resonance angiography (TOF-MRA) when there is a detectable signal of blood flow. In acute ischemic stroke patients, however, TOF-MRA does not show branching patterns with no detectable flow signal beyond the large vessel occlusion. Using a fusion image with TOF-MRA, a T2-weighed high-resolution vessel wall MRI or 3D cisternography acquired by a heavily T2-weighted MRI sequence can outline vessel anatomy ahead and behind the clot [7,8,9,10,11,12]. Flat panel detector or cone-beam CT angiographic images were obtained after an intra-arterial diluted contrast injection showing about 90% of excellent or good image quality and about 70% visualization of the distal portion to the occluded artery segment by using regrade flow [13,14].

CT is the imaging modality preferred over MRI as a routine imaging protocol to triage acute ischemic stroke patients given short scanning and reconstruction time. CTA is a powerful tool to detect accessible artery occlusion and find salvageable brain tissue on source images within the golden time. To our knowledge, this is the first approach leveraging NCT to outline the distal-to-thrombus segment of large vessel occlusions in cases of acute ischemic stroke, beyond detecting thrombus segments by hyperdense artery signs.

This retrospective single-center study based on the arterial visualization grade showed the potential feasibility of NCT and CTA in visualizing distal-to-thrombus segments prior to performing thrombectomy. We found that arterial tracing either by using visualization grade on NCT or CTA performs well for this purpose; among 150 patients in our study, 89 patients revealed a NCT grade of distal-to-thrombus segment above 4 (59.3%) and 66 patients revealed a CTA grade of distal-to-thrombus segment above 4 (44%). Interestingly, we found that arterial tracing of a distal-to-thrombus segment on NCT was equivalent to or better than that of CTA in 73% of our patients, and the mean visualization grade of a distal-to-thrombus segment on NCT was significantly larger than that of CTA. First, this finding may be due to better preservation of minute image contrast in NCT, which makes the arterial structures more discernable from the surrounding cisternal space. This is in contrast to the lower preservation in the CTA during iodine contrast enhancement. This tendency was more prominently shown in older subjects with age-related brain atrophy. In patients with wide cisternal space due to brain atrophy, the distal-to-thrombus segment arterial course tended to be more obviously visible. Secondly, it may be attributed to the reconstituted or collateralized flow, which is shown to be variable in incidence. When the collateral flow was good, the distal-to-thrombus branch tended to be more visible on CTA. The CTA grade in the distal-to-thrombus segment of the good collateral flow subgroup was higher than that of the poor collateral flow subgroup. In terms of distal-to-thrombus segment, the collateral score of the CTA-superiority subgroup (CTA_grade_ > NCT_grade_) was higher than that of the CTA-inferiority subgroup (CTA_grade_ ≤ NCT_grade_). This result was in accordance with the intra-arterial cone-beam CT angiographic studies [13,14], in that the visualization grade was significantly correlated with the collateral score. On the other hand, the NCT grade in the thrombosed segment of the poor collateral flow subgroup was significantly larger than that of the good collateral flow subgroup, likely due to slow flow and hyperdense arterial sign. Third, in addition to the variable collateral flow pattern, thrombus permeation of the iodine contrast medium might affect the visualization of the distal-to-thrombus arterial course. In our study, the CTA grade in the distal-to-thrombus segment of the permeation subgroup was higher than that of the non-permeation subgroup, suggesting that the CTA grade of the distal-to-thrombus segment is related to the CTA grade of the thrombosed segment in the permeation subgroup. From the dynamic CTA data of acute ischemic stroke patients, thrombus permeability was estimated on the assumption that there were numerous channels within the thrombus [25]. Thrombus enhancement on CTA may suggest a biomarker predictor of certain etiology, such as identifying thrombi with a higher fibrin-to-platelet fraction and a lower erythrocyte proportion [26,27,28]. In this study, however, no differences in arterial visualization grade were found between cardiac and non-cardiac emboli, regardless of the mode of imaging (NCT or CTA) or the location of the segment (thrombosed or distal-to-thrombus segment).

On the other hand, in the task of predicting the presence of occlusion segments, CTA was significantly better than NCT, as suggested by the receiver operating characteristic curve. Although NCT was better at visualizing the distal-to-thrombus arterial course, the detection of thrombosed segments was more reliable on CTA. In this study, CTA outperformed NCT in determining the occluded hemisphere regardless of the thrombosed segment and distal-to-thrombus arterial course.

Hence, the combined interpretation of NCT and CTA might be beneficial in outlining the distal-to-thrombus arterial course, as shown in 17 cases (11%) of this study where the distal-to-thrombus segment visualization was improved as compared to the NCT or CTA interpretation alone. Piecing arterial branching patterns together based on a comprehensive reading of both NCT and CTA provides anatomic details and may be useful for effective treatment strategies.

We acknowledge several limitations of our study; first, the retrospective study design of a single institution would have led to a selection bias. Our findings warrant further validation in a larger prospective multi-center study. Second, scoring arterial visualization grades was performed by consensus of an experienced interventional neuroradiologist and a trainee with openness to DSA findings. This may have exaggerated the scoring performance of the CT modality in this study. It would be necessary to conduct a further inter-rater variability study including the interpretation times required for multiple readers with various spectrums of experience. Although the combined interpretation of NCT and CTA may be more challenging for a beginner, it might be a usual process for an experienced operator who, before performing a thrombectomy, assesses the visualization grade in a real field. Interestingly, we noted the rapid learning of a trainee in the process of consensus arterial visualization grading. Third, this study included MIP reconstruction from cerebral CTA, brain perfusion CT, and even the carotid CTA. Intra-arterial injection cone-beam CT was not attempted to compare the visualization grade. The impact of different scanning parameters and reconstructive protocols should be considered in future studies. Fourth, multiplanar reconstruction of the NCT and CTA may provide a better depiction of the thrombosed and distal-to-thrombus segments. However, this proof-of-concept study assessed the sagittal plane alone for simplicity of analysis [29]. M2 or M3 branching patterns surrounded by Sylvian cistern are more discernable at the sagittal plane than other planes. The effect of a combination of three planes to augment predictive value should be assessed in future studies. Fifth, we did not classify the MCA branching pattern or determine a target dominant branch among anatomical MCA variations [19,20,21]. Sixth, the current study tested the feasibility of arterial visualization grading only. Future investigations should relate this new finding to major clinical outcomes such as recanalization rates and patient outcomes.

## 5. Conclusions

Tracing the arterial course and piecing branching patterns together in distal-to-large-vessel occlusion of acute ischemic stroke patients is feasible based on the routine pre-interventional imaging modality of NCT and CTA. This approach may provide timely guidance during endovascular thrombectomy, obviating the need for more time-consuming MRI, and reducing the potential risk related to endovascular device navigation.

## Figures and Tables

**Table 1:** Demographics and baseline characteristics of patients (*n* = 150).

**Patient Characteristics**	**Numbers (%)**
**Total number (%)**	150 (100)
**Ages (years)**	
Median	73
Range	20–95
**Gender**	
Male	84 (56.0)
Female	66 (44.0)
**CT protocol**	
Cerebral artery CTA	82 (54.7)
Perfusion CT and CTA	66 (44.0)
Carotid artery CTA	2 (1.3)
**Occlusion site**	
ICA terminus	15 (10.0)
ICA and MCA tandem occlusion	48 (32.0)
MCA M1 segment	75 (50.0)
MCA M2 segment	12 (8.0)
**NIHSS (National Institutes of Health Stroke Scale)**	
Initial NIHSS	13 ± 5
Discharge NIHSS	10.5 ± 9.3
**mRS (Modified Rankin Scale)**	
Initial mRS	0.2 ± 0.8
Discharge mRS	4 ± 1.4
**Underlying disease**	
Hypertension	84 (56)
Diabetes mellitus	36 (24)
Hyperlipidemia	52 (34.7)
Cardiac disease	63 (42)

CTA, computed tomographic angiography; ICA, internal carotid artery; MCA, middle cerebral artery.

## Data Availability

The data presented in this study are available on request from the corresponding author.

## Supplementary Materials

The following supporting information can be downloaded at: https://www.mdpi.com/article/10.3390/jcm12124051/s1, Figure S1: Collateral grading in DSA. (A) A 52-year-old male with left MCA M2 occlusion with poor collateral. (B) An 85-year-old male with right MCA M2 occlusion with good collateral. DSA, digital subtraction angiography; MCA, middle cerebral artery. Figure S2: NCT and CTA performance to detect the occlusion side. The AUC of the CTA grade of thrombosed segment and that of distal-to-thrombus segment was 1.00 and 0.897, respectively. The AUC of the NCT grade of thrombosed segment and that of distal-to-thrombus segment was 0.498 and 0.535, respectively. AUC, area under curve; NCT, non-contrast computed tomography; CTA, computed tomographic angiography. Table S1: Definition of arterial visualization grade. Table S2: Definition of collateral score. Table S3: Arterial visualization grading improvements after combined interpretation of NCT and CTA.

## Abbreviations

NCT = non-contrast computed tomography; CTA = computed tomographic angiography; DSA = digital subtraction angiography; TICI = thrombolysis in cerebral infarction.
