# Supplementary material for: Piecing Arterial Branching Pattern Together from Non-Contrast and Angiographic Brain Computed Tomography before Endovascular Thrombectomy for Acute Ischemic Stroke
Source: J Clin Med. 2023 Jun 14;12(12):4051. doi: 10.3390/jcm12124051 (PMC10298971; doi:10.3390/jcm12124051)
Supplement: Supplementary file 1 [file jcm-12-04051-s001.zip › jcm-2334577-Supplementary Materials.pdf]

Journal of
Clinical Medicine

MDPI

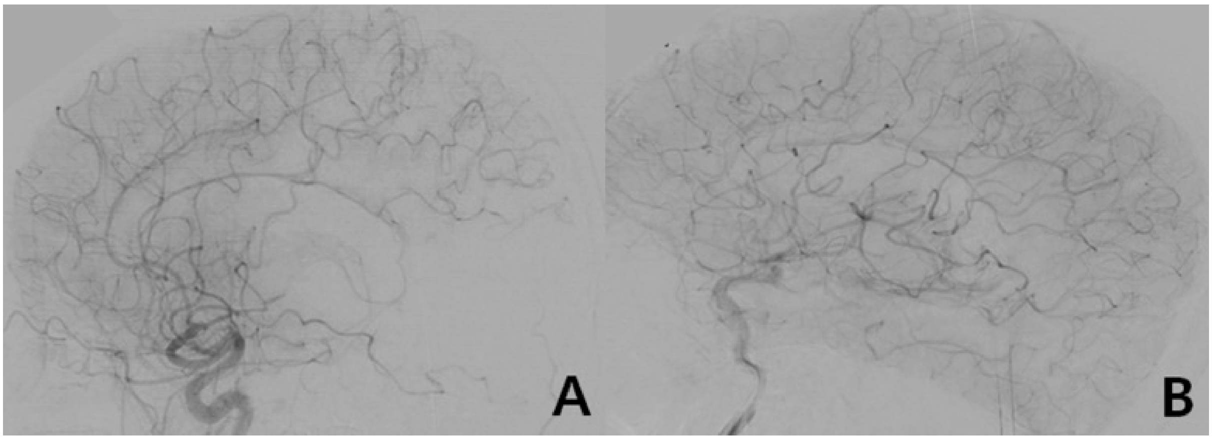

Supplementary Figure S1. Collateral grading in DSA. (A) A 52-year-old male with left MCA M2 occlusion with poor collateral. (B) An 85-year-old male with right MCA M2 occlusion with good collateral. DSA, digital subtraction angiography; MCA, middle cerebral artery.

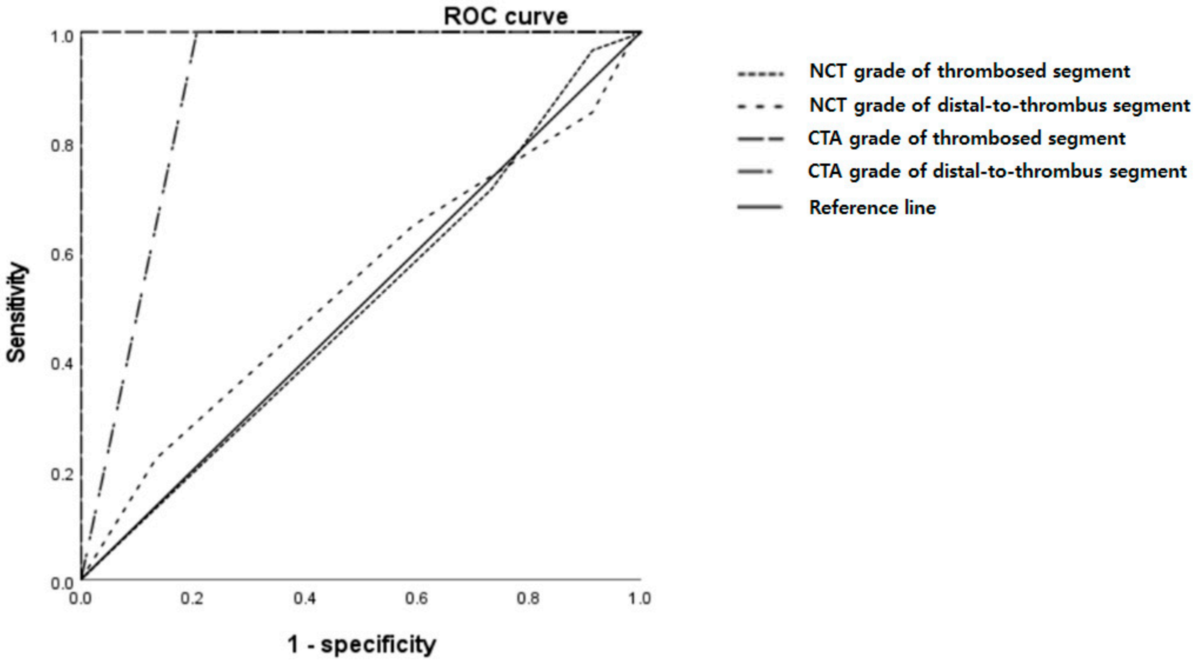

Supplementary Figure S2. NCT and CTA performance to detect the occlusion side. The AUC of the CTA grade of thrombosed segment and that of distal-to-thrombus segment was 1.00 and 0.897, respectively. The AUC of the NCT grade of thrombosed segment and that of distal-to-thrombus segment was 0.498 and 0.535, respectively. AUC, area under curve; NCT, non-contrast computed tomography; CTA, computed tomographic angiography.

Supplementary Table S1: Definition of arterial visualization grade

Arterial Visualization Grade	Definition
1	The vessel course is completely unpredictable
2	The vessel course is poorly predicted. e.g., only a small portion of the M2 segment of MCA is seen
3	The vessel course is partially predicted. e.g., only one branch of the M2 segment of MCA is seen completely, or just a small portion of all MCA branches is seen
4	The vessel course is predicted well. e.g., one branch of the M2 segment of MCA is fully visible and just a small portion of the other branches are predicted
5	The vessel course is predicted very well or all M2 segments of the MCA are predicted

MCA, middle cerebral artery

Supplementary Table S2: Definition of collateral score

Collateral Score	Definition
0	No collateral flow to the ischemic area
1	Slow collateral flow to ischemic regions with persistent perfusion defect
2	Fast collateral flow to ischemic regions with persistent perfusion defect
3	Slow collateral flow to ischemic regions without persistent perfusion defect
4	Fast collateral flow to ischemic regions without persistent perfusion defect

Supplementary Table S3: Arterial visualization grading improvements after combined interpretation of NCT and CTA

NCT grade of distal-to-thrombus segment	CTA grade of distal-to-thrombus segment	Visualization grade after comprehensive reading	N (%)
3	2	4	1 (5.9)
3	3	4	10 (58.8)
3	3	5	1 (5.9)
4	4	5	5 (29.4)

NCT, non-contrast computed tomography; CTA, computed tomography angiography; N, number
